# The interaction effect between gender and profession in posttraumatic growth among hospital personnel

**DOI:** 10.1017/S1463423620000377

**Published:** 2020-09-24

**Authors:** Yaira Hamama-Raz, Menachem Ben-Ezra, Haim Bibi, Muhareb Swarka, Renana Gelernter, Ibrahim Abu-Kishk

**Affiliations:** 1 School of Social Work, Ariel University, Ariel, Israel; 2 Pediatric Intensive Care Unit, Shamir (Assaf Harofeh) Medical Center, Zerifin 70300 Israel (affiliated to the Sackler Faculty of Medicine, Tel-Aviv University); 3 Internal Medicine Department “F”, Shamir (Assaf Harofeh) Medical Center, Zerifin 70300 Israel (affiliated to the Sackler Faculty of Medicine, Tel-Aviv University)

**Keywords:** gender, nurses, physicians, posttraumatic growth, profession

## Abstract

**Aim:**

To explore if there is an interaction effect between gender (men and women) and profession (nurses and physicians) in posttraumatic growth (PTG).

**Background:**

PTG is defined as a positive psychological change experienced as a result of struggling with highly challenging life circumstances. It may take the form of improved self-image, a deeper understanding of self, increased spirituality, and/or enhanced interpersonal relationships. Gender and profession were found separately to be associated with PTG, but to date were not examined under interaction effect.

**Methods:**

We employed a cross-sectional study conducted in the tertiary medical center in Israel using a convenience sample. One hundred and twenty-eight nurses and seventy-eight physicians gave their consent and agreed to fill out self-report questionnaires regarding personal and professional data and PTG Inventory.

**Findings:**

The correlation matrix revealed that being a woman was associated with higher PTG total scale (*r* = 0.242; *P* ≤ 0.001) and its subscales except for spiritual change that showed no evidence of statistical effect. Similar pattern was found for being a nurse with PTG total scale (*r* = 0.223; *P* ≤0.001) and its subscales except for relating to others that showed no evidence of statistical effect. However, the interaction effect revealed that among men, there was no difference in the level of PTG and its subscales based on profession (Physicians men = 62.54 (20.82) versus Nurses men = 60.26 (22.39); *F* = 9.618; *P* = 0.002). Among women, nurses had a significantly higher scores in PTG (Physicians women = 61.81 (18.51) versus Nurses women = 73.87 (12.36); *F* = 9.618; *P* = 0.002) and its subscales in comparison to physicians except for subscale relating to other.

**Conclusions:**

Our findings suggest implications for research and practice namely exploring PTG among nurses and physicians would benefit from applying interaction effect of gender and profession. For practice, advocating PTG within the health care organization is needed to be tailored with gender and professional sensitivity.

## Introduction

Health care professions, especially physicians and nurses, are among the occupational group that exposed to higher work-related stress, results from their responsibility for health and well-being of patients, and their continues exposure to death, injury, or victims (eg, Ogińska-Bulik, 2006; Stucky *et al.*, 2009). These factors triggered psychological reactions which were documented by a large body of literature in two ways that can co-exist: the first relates to negative reactions namely, secondary traumatic stress, burnout, and compassion fatigue (eg, Cocker and Joss, 2016; El-Bar *et al.*, 2013; Hamama *et al.*, 2019), while the second composed of positive reactions namely, posttraumatic growth (PTG) and satisfaction fatigue (eg, Hamama-Raz and Minerbi, 2019; Manning-Jones *et al.*, 2016; 2017; Shoji *et al.*, 2014). In the current research, we aimed to focus on the positive reactions of work-related stress in health care professions namely PTG.

### Posttraumatic growth

PTG relates to the salutogenic aspects of human experience in the face of adverse event (Höltge *et al.*, 2018); it was defined by Tedeschi and Calhoun (2004) as “the experience of positive change that occurs as a result of the struggle with highly challenging life crises” (p. 1). PTG is manifested in five domains, including an increased appreciation for life in general, more meaningful interpersonal relationships, an increased sense of personal strength, changed priorities, and a richer existential and spiritual life (Tedeschi and Calhoun, 1996; 2004). Thus, those who have experience of challenging life events may feel as stronger and better able to deal with difficult events in the future and may also experience a greater sense of intimacy and belonging. Additionally, they may gain a greater sense of purpose and appreciation for life, and new priorities regarding what is most important. In line with this notion, Malhotra and Chebiyan’s overview (2016) stated that PTG can be placed within either of two descriptions: in the first, PTG is presented as a by-product of attempts to cope with life-changing traumatic events, where these attempts manifest in the development of a life narrative and wisdom. In the second description, PTG is presented as a coping strategy which may include construal of meaning, meaning-making coping process, interpretative process, or form of self-enhancing appraisal or positive illusion with an adaptive function for psychological adjustment. Actually, according to the Conservation of Resources theory (Hobfoll, 1989; 2011), individuals seek to build and retain resources as a result from actual or threatened loss of resources after coping with stressful circumstances. Resource gains can help to offset the impact of resource losses. Given this, PTG is an important source of resource gain after stressful events that could help to buffer against resource loss caused by work-related stressors. Given this notion, in the current study, we assessed PTG as a by-product result of health care providers’ (ie, physicians and nurses) exposure to a variety of work-related stress.

### PTG among health care professions

Previous researches which explored the link between profession affiliation and PTG among health care provider revealed that PTG is a very common phenomenon in all health care providers (ie, therapists, social workers, counsellors, nurses, and physicians – for a review see Manning-Jones *et al.*, 2015). Nevertheless, when comparing nurses and physicians’ PTG level, most of the studies indicated that nurses reported on higher level of PTG than physicians (eg, Manning-Jones *et al.*, 2016; Shiri *et al.*, 2008; Taubman Ben-Ari and Weintroub, 2008). Only Rodríguez-Rey *et al.* (2017) found among professionals working in pediatric intensive care personnel and personnel working at other pediatric wards that PTG was not different among physicians, nurses and nursing assistants. Possible explanation which was suggested by the researchers referred to the difference in the character of their work. Specifically, nurse’s role involves much closer contact with patients and more intimate involvement in their physical, physiological, and mental needs (Taubman Ben-Ari and Weintroub, 2008). Additionally, nurses’ responsibilities regarding patients’ care required them to spend long time with them, whereas physicians meet the patients for shorter periods of time during their daily rounds. In line with this notion, Moriel *et al.* (2017) show through a qualitative research via focus groups conducted with practicing nurses and physicians that physicians perceive nurses as the patients’ advocates, having much closer interaction with patients “patients see the nurse as liaison and caregiver—and one of nurses’ primary roles is that of patient assessor” (p. 8). Nurses concurred on three main physician roles: prescriber, decision-maker, and educator. Another explanation which may account for the profession’s differences in PTG level was related to gender as nursing has traditionally been and remains a female-dominated profession in Western countries (Kouta and Kaite, 2011; Solbrække *et al*., 2013).

### PTG and gender

The scientific literature that explored PTG among women and men indicate that women show higher levels of PTG than men who experience similar traumatic events (eg, Helgeson *et al.*, 2006; Vishnevsky *et al.*, 2010). According to Vishnevsky *et al*.’s (2010) meta-analysis, possible explanation for this difference refers to women’s tendency to engage in deeper thought than men. The tendency to think deeply on constructive issues, such as an increased awareness of personal strengths or an appreciation of the importance of social connections, has been suggested as a mechanism leading to greater reports of PTG.

Nevertheless, in references to health care professional – previous studies revealed no gender differences. Specifically, in Manning-Jones *et al*.’s studies (2016; 2017) who explored differences between several distinct groups of health professionals including nurses and physicians, no significant differences emerged between women and men with regard to PTG. Likewise, Rodríguez-Rey *et al.* (2017) who investigated PTG among staff in pediatric intensive care units in comparison to other pediatric units revealed that PTG scores did not differ by gender. Also, Kang *et al.* (2018) reported no gender differences with PTG among nurses and physicians and other ambulance workers in Chania.

### The current research

In the current study, we sought to focus on PTG among physicians and nurses and to explore the interaction effect between gender and profession in PTG – two factors which were found separately to be associated with PTG (eg, Kang *et al.*, (2018; Manning-Jones *et al.*, 2015; Manning-Jones *et al.*, 2016; Rodríguez-Rey *et al.*, 2017; Taubman Ben-Ari and Weintroub, 2008), but to date were not examined under interaction effect. We expected that the interaction between these two factors will exhibit much more detailed picture concerning PTG among hospital personnel.

## Methods

### Procedure

The study was conducted in a tertiary hospital in Israel with 750 physician and nurses. The sampling used was a convenience sample by approaching directly the hospital personnel (physicians and nurses) and asking them to answer a self-repot questionnaires assuring confidentiality and explaining the voluntary nature of their participation. Data collection took place between 15/April and 1/June/2019. The estimated sample size using 5% margin of error, 90% confidence interval yielded a needed sample of 200 participants. Our final sample size was 206. Participants were recruited from every ward of the hospital, and the only inclusion criterion was a licensed physician or nurse.

### Ethics statement

The Institutional Review Board and Ethics Committee of the hospital approved the study protocol. Participants were informed that their participation is entirely voluntary and that data obtained from the survey would be analyzed in an anonymous format.

### Measures

Participants completed the following self-report questionnaires:

***Personal and professional data*** – which served as the independent variables for this study. We inquired regarding gender, age, marital status, profession, education, and degree of religiosity. In addition, participants were asked to indicate their years in the profession, their years in the current department, employment scope (part-time employment percentage or full-time employment). Self-rated health was assessed with a single question: “In general, how do you rate your health?” The scale ranged from 1 to 4 (1 = “*bad”* to 4 = “*excellent”*). This measure was found to be valid and highly associated with objective indicators of health (Benyamini *et al.* 2003).

***PTG** –* the dependent variable for this study. It was measured by the PTG Inventory (PTGI; Tedeschi and Calhoun, 1996; Original Cronbach’s alpha was 0.90), Hebrew version (Laufer and Solomon 2006; Cronbach’s alpha of the Hebrew version was 0.94) in order to assess positive changes after negative life events. This 21-item scale is composed of one global score of PTG and five subscales. Each subscale is measuring a different facet of post traumatic growth: New Possibilities (5 items), Relating to Others (7 items), Personal Strength (4 items), Spiritual Change (2 items), and Appreciation of Life (3 items). The answers were given in a six-point Likert response format ranging from *I did not experience this change as a result of my crisis* (scored 0), to *I experienced this change to a very great degree as a result of my crisis* (scored 5). The PTGI has no cut-off values. The higher the score, the more PTG the person has This is considered positive as it means the person shows more affinity to new possibilities, higher relations to others, more personal strength, more positive spiritual change, and higher appreciation of life.

Cronbach’s alpha and basic descriptive statistics for the current study were 0.94 for total PTGI score (mean score = 67.33; SD = 18.15) and for each subscale: New Possibilities (α = 0.86; mean score = 15.44; SD = 5.32), Relating to Others (α = 0.87; mean score = 22.30; SD = 6.28), Personal Strength (α = 0.82; mean score = 14.20; SD = 3.70), Spiritual Change (α = 0.61; mean score = 5.13; SD = 2.54), and Appreciation of Life (α = 0.82; mean score = 10.08; SD = 3.28).

## Data analysis

Before commencing the analytic plan, we have checked for data integrity and missing values. One participant had missing values and has been omitted from the study leading to a final sample of 206 participants.

The analytic plan had two stages: First: we present a simple correlation matrix for the study variables. Second, we used multivariate analysis of covariance (MANCOVA) with gender (men/women) and position (nurse/physician) as independent variables. Age, marital status, years of experience in medicine, years of experience in the department, and percentage of employment served as covariates. The dependent variables were the total PTGI score and the five subscales namely: New possibilities, Relating to others, Personal strength, Spiritual change, and Appreciation of life. The analysis was accompanied by effect size as measured by partial eta square. Interaction effects were accompanied by corresponding figures. Data were analyzed using IBM SPSS ver. 25.

## Results

The study was conducted in tertiary hospital in Israel among health care providers, namely physicians and nurses. The mean age was 41.48 (SD = 17.70), 62% of the participants were women (*n* = 130), 61.8% of the participants were nurses (*n* = 128), and the rest were physicians. Among women, the profession distribution was 97 nurses and 32 physicians. Among men, the position distribution was 46 physicians and 31 nurses. About 75.8% of the participants reported being in a committed relationship (*n* = 157). The mean years of experience in medicine was 12.83 (SD = 10.68), and the mean years of experience in the department was 9.40 (SD = 9.78). The participants employed in a full-time job was 165 (79.7% of the sample), and the rest in varied capacity ranging from 40% to 95%.

The correlation matrix revealed that being a woman was associated with higher PTG total scale and its subscales except for Spiritual change that was not found to be significant. Similar pattern was found for being a nurse with PTG total scale and its subscales except for Relating to others that was not found to be significant. For more information, see Table 1.

**Table 1.** Correlation matrix of the study variables (*N* = 206)

	1	2	3	4	5	6	7	8	9	10	11	12	13
1. Age													
2. Gender	0.096												
3. Profession	0.051	0.348***											
4. Marital status	0.178**	0.103	−0.022										
5. Years of medical experience	0.537***	0.117	0.130	0.218***									
6. Years of experience in the current department	0.398***	0.151*	0.172*	0.129	0.665***								
7. Work capacity	−.074	−0.158*	−0.093	−0.103	−0.229***	−0.191**							
8. PTG – RTO	.023	0.257***	0.115	−0.007	0.046	−0.009	0.025						
9. PTG – NP	−.018	0.199**	0.261***	−0.094	−0.011	−0.045	0.012	0.743***					
10. PTG –PS	−.028	0.205**	0.210**	−0.036	0.051	0.037	0.056	0.729***	0.758***				
11. PTG – SC	−.019	0.121	0.233**	−0.003	−0.034	0.002	−0.022	0.506***	0.576***	0.440***			
12. PTG – AOL	−.033	0.224***	0.178**	−0.017	0.061	−0.065	−0.063	0.706***	0.812***	0.735***	0.512***		
13. PTG – TOT	−.012	0.242***	0.223***	−0.043	0.029	−0.023	0.017	0.906***	0.915***	0.868***	0.661***	.867***	

PTG = posttraumatic growth; PTG – RTO = Relating to Others, PTG – NP = New Possibilities, PTG – PS = Personal Strength, PTG – SC = Spiritual Change, PTG – AOL = Appreciation of Life, PTG – TOT = Total.

*Note:* Gender Coded as 1 = Men; 2 = Women. Profession coded as 1 = Nurses; 2 = Physicians.

* = *P* ≤ 0. 005; ** = *P* ≤ 0.01; *** = *P* ≤ 0.001;

Following the correlation matrix, the MANCOVA results revealed a significant main effect of gender regarding PTG scores and its subscales except for spiritual change. *F* statistics ranged from 4.146 to 12.335, and *P*-value was ranged from 0.043 to 0.001. Effect size as measured partial η^2^ ranged from 0.021 to 0.059. The main effect of profession regarding PTG scores and its subscales revealed only two significant results namely new possibilities (*F* = 7.634; *P* = 0.006; partial η^2^ = 0.037) and spiritual change (*F* = 7.426; *P* = 0.007; partial η^2^ = 0.036). In order to be concise, the mean scores and standard deviation for the PTGI and its subscales are presented in Table 2.

**Table 2.** Mean scores and standard deviations of the posttraumatic growth inventory and its subscales (*n* = 206)

Scale	Sex	Profession	Mean	SD
Total PTGI Score	Men	Physician	62.5435	20.82809
		Nurse	60.2581	22.38745
	Women	Physician	61.8125	18.51144
		Nurse	73.8660	12.35637
Relating to others	Men	Physician	20.8913	7.27928
		Nurse	19.1935	7.63509
	Women	Physician	22.1250	6.47950
		Nurse	24.0619	4.55250
New possibilities	Men	Physician	13.9348	5.66236
		Nurse	14.2581	6.67317
	Women	Physician	13.4063	5.07911
		Nurse	17.2990	4.02380
Personal strength	Men	Physician	13.3478	4.50292
		Nurse	13.0323	4.30878
	Women	Physician	13.0313	3.67629
		Nurse	15.3918	2.62820
Spiritual change	Men	Physician	4.6739	2.76530
		Nurse	4.8065	2.63843
	Women	Physician	3.9688	2.26451
		Nurse	5.8557	2.29124
Appreciation of life	Men	Physician	9.3913	3.70272
		Nurse	8.7419	3.57741
	Women	Physician	9.2500	3.15206
		Nurse	11.1134	2.71522

**Table 3.** Results of the MANCOVA (*N* = 206)

Source	Dependent variable	Type III sum of squares	df	F	Sig.	Partial Eta squared
Age	Relating to others	2.147	1206	0.058	0.810	0.000
	New possibilities	1.677	1206	0.067	0.796	0.000
	Personal strength	18.181	1206	1.447	0.230	0.007
	Spiritual change	0.117	1206	0.019	0.890	0.000
	Appreciation of life	13.090	1206	1.341	0.248	0.007
	Posttraumatic growth	122.911	1206	0.418	0.519	0.002
Marital status	Relating to others	15.715	1206	0.426	0.515	0.002
	New possibilities	62.606	1206	2.499	0.116	0.013
	Personal strength	9.255	1206	0.737	0.392	0.004
	Spiritual change	0.001	1206	0.000	0.989	0.000
	Appreciation of life	6.614	1206	0.677	0.411	0.003
	Posttraumatic growth	320.926	1206	1.091	0.298	0.006
Years of experience in profession	Relating to others	34.566	1206	0.936	0.334	0.005
	New possibilities	5.166	1206	0.206	0.650	0.001
	Personal strength	10.272	1206	0.818	0.367	0.004
	Spiritual change	4.780	1206	0.784	0.377	0.004
	Appreciation of life	40.058	1206	4.103	0.044	0.020
	Posttraumatic growth	254.385	1206	0.865	0.354	0.004
Years of experience in the current department	Relating to others	60.779	1206	1.646	0.201	0.008
	New possibilities	61.636	1206	2.460	0.118	0.012
	Personal strength	2.457	1206	0.196	0.659	0.001
	Spiritual change	0.095	1206	0.016	0.901	0.000
	Appreciation of life	68.759	1206	7.044	0.009	0.035
	Posttraumatic growth	690.121	1206	2.346	0.127	0.012
Percentage of employment	Relating to others	62.473	1206	1.692	0.195	0.009
	New possibilities	22.751	1206	0.908	0.342	0.005
	Personal strength	44.843	1206	3.570	0.060	0.018
	Spiritual change	0.322	1206	0.053	0.819	0.000
	Appreciation of life	0.000	1206	0.000	0.997	0.000
	Posttraumatic growth	479.569	1206	1.630	0.203	0.008
Gender	Relating to others	455.534	1206	12.335	0.001	0.059
	New possibilities	103.872	1206	4.146	0.043	0.021
	Personal strength	62.420	1206	4.970	0.027	0.025
	Spiritual change	1.954	1206	0.320	0.572	0.002
	Appreciation of life	65.247	1206	6.684	0.010	0.033
	Posttraumatic growth	2311.952	1206	7.859	0.006	0.038
Profession	Relating to others	1.247	1206	0.034	0.854	0.000
	New possibilities	191.240	1206	7.634	0.006	0.037
	Personal strength	40.056	1206	3.189	0.076	0.016
	Spiritual change	45.285	1206	7.426	0.007	0.036
	Appreciation of life	17.740	1206	1.817	0.179	0.009
	Posttraumatic growth	1046.726	1206	3.558	0.061	0.018
Gender × profession	Relating to others	174.508	1206	4.725	0.031	0.023
	New possibilities	199.643	1206	7.969	0.005	0.039
	Personal strength	98.730	1206	7.860	0.006	0.038
	Spiritual change	40.526	1206	6.646	0.011	0.033
	Appreciation of life	74.159	1206	7.597	0.006	0.037
	Posttraumatic growth	2829.293	1206	9.618	0.002	0.047

* = *P* ≤ 0.05; ** = *P* ≤ 0.01; *** = *P* ≤ 0.001.

**Figure 1.**
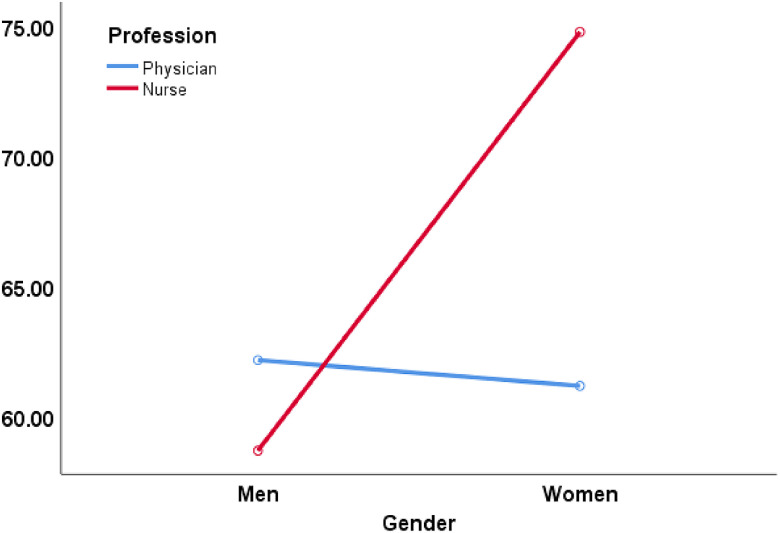
Interaction of gender × position regarding PTG total score.

Contrary to main effects, we found a pronounced and significant interaction effect (Gender × Profession) regarding PTG scores and its subscales. *F* statistics ranged from 4.725 to 9.618, and *P*-value was ranged from 0.031 to 0.002. Effect size was measured by partial η^2^ ranged from 0.023 to 0.047. It seems that a pronounced interaction effect between gender and profession was across the board, meaning this interaction effect was found in the general PTG score and in each of its subscales. Among men, there was no difference in the level of PTG and its subscales based on profession. However, among women, nurses had a significantly higher scores in PTG and its subscales in comparison to physicians except for PTG subscale Relating to others (this result albeit significant in the MANCOVA was found as non-significant in a t-test among women split based on profession). See Figure 1 for the PTG interaction. PTG subscales figures are presented in the online supporting material.

Another interesting results revealed that years of experience in the profession (*F* = 4.103; *P* = 0.044; partial η^2^ = 0.020) and years of experience in the current department (*F* = 7.044; *P* = 0.009; partial η^2^ = 0.035) were associated significantly with PTG subscale Appreciation of life (Table 3).

## Discussion

The present study aimed to explore if there was an interaction effect between gender (women and men) and profession with the assessment of PTG among nurses and physicians in a tertiary care hospital. The results reveled that PTG among nurses and physicians is better to be assessed in light of this interaction effect, and not as separate factors that are linked to PTG. Specifically, our main findings show that among men, PTG was not different in male nurses and male physicians. However, when it comes to women, nurses exhibited higher scores of PTG and its subscales (except for PTG subscale relating to others) in comparison to women physicians. These findings were not previously known, as previous studies did not explore the interaction effect of gender and profession. Thus, we cannot suggest reinforcement or disapproving of the current results.

Yet, possible explanation may be related to the differences between women physicians in comparison to their male counterparts. According to Robinson (2003), women physicians may lack role models, face challenges of dual-career couples, have to reconcile with a finite number of years for childbearing, face lack of parity in salaries, receive a lower number of promotions to leadership positions, and experience higher rates of sexual harassment. Moreover, Roter and her colleagues (2002) found in their meta-analytic review about medical communication among physicians that women physicians engage in more communication that can be considered patient-centered and have longer visits than their male colleagues. The aforementioned factors may affect their work-related stress and in turn their PTG experience. In line with this notion, previous studies showed that the higher the distress among health care providers, the lower their personal growth (eg, Hagenaars and van Minnen, 2010; Măirean, 2016). Nevertheless, our results did not reveal profession differences with PTG subscale of Relating to others between women nurses versus women physicians. According to Tedeschi and Calhoun (1996; 2004), this subscale refers to recognition of one’s vulnerability which can lead to more willingness to accept help, more expressiveness, and increased self-disclosure. The individual may perceive a higher emotional connection with others, as well as a feeling of closeness in interpersonal relationships. Possible explanation may stem from women’s gender-specific socialization messages regarding roles, emotions, and cognitions. Sociologists describe the sex role socialization as “instrumental” for men and “expressive” for women. Expressive socialization includes learning to nurture, to be affiliative, and to be sensitive to needs of others (Strasen, 1992). Given that, it may be reasonable to assume that this factor of PTG will not be different in both professions as it in the essence of being a woman.

With regard to the absence of difference with PTG scores among male nurses versus male physicians, it may be that masculine attributes are more dominant than profession. In line with this notion, Wu *et al*. (2015) suggested that male nurses distanced themselves from the more emotive aspects of care as a form of protection, objectively providing patient and family care. Likewise, Hollup (2014) noted that male nurses may find it difficult to assume the caregiver role while negotiating a feminized environment because it disrupts the gender normative view of masculinity. Moreover, according to Roter and Hall, (2015), women physicians exhibit more emotional response to their patients in comparison to male physicians, by talking more about emotions, and by expressing empathy and concern. These findings confirmed the role gender attributes as being above the profession’s differences.

Concerning the typical investigation of the link between profession and PTG, our results confirmed previous researches (eg, Manning-Jones *et al*., 2016; Shiri *et al.*, 2008; Taubman Ben-Ari and Weintroub, 2008) showing that nurses reported on higher level of PTG than physicians. Possible explanation which was also suggested by the researchers (eg, Moriel *et al.*, 2017; Taubman Ben-Ari and Weintroub, 2008) referred to the difference in the character of their work. Specifically, nurse’s role involves much closer contact with patients and more intimate involvement in their physical, physiological, and mental needs. As such, they might be exposed to higher work-related stress but at the same time may enjoy from high PTG due to the positive link between posttraumatic stress and PTG (eg, Hamama-Raz and Minerbi, 2019). Another possible explanation might stem from the availability and the enactment of peer social support among nurses in comparison to physicians. According to Duffy *et al.* (2015), nurses may have more opportunities to share and communicate work stressors due to the presence of more co-workers from the same profession. However, among physicians the opportunities to share their emotions experienced after exposure to work-related stressors available only with few co-workers present (Bruce *et al.*, 2005).

With regard to the link between gender and PTG, our findings revealed that being a woman was associated with higher PTG total scale and its subscales except for Spiritual change. This result was not found in previous researches in references to health care professional (eg, Kang *et al.*, 2018; Manning-Jones *et al*., 2016, 2017; Rodríguez-Rey *et al.*, 2017); however, among the general population (men and women who experience similar traumatic events), indeed women show higher levels of PTG than men (eg, Helgeson *et al.*, 2006; Vishnevsky *et al.*, 2010). Possible explanation may stem from Vishnevsky *et al.*’s (2010) meta-analysis that suggests to relate to women’s tendency to engage in deeper thought than men. This tendency may increase awareness of personal strengths or an appreciation of the importance of social connections and has been suggested as a mechanism leading to greater reports of PTG (Vishnevsky *et al.*, 2010). In line with this notion, the Theory of Cognitive Adaptation (Taylor, 1983) posits that humans cope with threats in their lives by creating a set of positive illusions, which serve to protect their psychological health. As such, Czajkowska (2017) claimed that “These positively slanted cognitions are not considered delusional or inaccurate but rather represent a sign of mental as they create space for hope, personal growth, and flexibility” (p. 1). With the aforementioned, it might be that woman’s tendency to engage in deeper thought enables to experience higher PTG.

Finally, our results revealed that participants’ years of experience in the profession and years of experience in the department were linked positively with PTG subscale – Appreciation of life. Tedeschi and Calhoun (1996; 2004) noted that due to the cognitive reconstruction, the individual’s priorities in life change and one experiences a greater appreciation of life in general and for the “smaller things” in life. In line with this notion, it might be that through the seniority of years in their profession, nurses and physicians were able to identify desirable elements of their existence that their patients are missing, as well as experiences that they fortunate to avoid. As such they appreciate much more the simple facets of their life (Hyatt-Burkhart, 2014).

### Limitations

The above discussion should be considered within several limitations. First, the present study is based on cross-sectional data; that is, the participants were asked to respond one single time, absent longitudinal follow-up, which did not allow to draw conclusions about causality. Thus, future studies recommended to employ longitudinal design that may provide indications of the nature of changes in health care providers’ PTG over time. Second, the sample is based on a single medical center, which may limit the potential generalizability of the results. Third, the Spiritual change had low reliability index meaning that the results of this subscale should be interpreted with cautious. Fourth, we may have a sampling bias as we used a convenience sampling in our study. Fifth, in a large sample, most correlations in a correlation matrix will show evidence of a statistical effect. Hence, these results should be interrupted with cautious. However, in order to overcome this, we have used the effect size in the MANCOVA procedure in order to reduce this bias.

Future studies might replicate the present one at other medical centers in other medical systems. Finally, the study relies on self-reports and thus might suffer from a social desirability bias.

### Implications

Taking into account the findings, this study carries implications in both research and clinical practice in several directions. With regard to research implication, the study results suggest to explore PTG among health care providers according to interaction effect of gender (women and men) with profession (nurses and physicians), as evaluating each factor separately might provide only partial findings regarding PTG. Considering the practical implications, the study’s results reinforce the need for tailoring intervention programs aimed at increasing PTG through gender and profession sensitivity, and not by gender or professional as a single factor. This can be achieved by encouraging nurses and physicians to take action (according the Action-Focused Growth model – Hobfoll *et al.*, 2007) in searching in line with their gender role a variety of sources of meaning, including defining their personal values, finding new goals, and perhaps, revising their life priorities. All of these are different aspects of the PTG process which should be translated into personal and social actions in order to renew feelings of competency (Hobfoll *et al.*, 2007) based on the interaction effects of gender and profession (See also Templeton *et al.*, 2019).

### Conclusions

The current study highlights the need for a more cohesive view of PTG among hospital workers rather than the traditional binary perception of PTG from either gender or profession. Future studies evaluating the interaction effect of gender with profession in PTG are warranted (among other professions), in order to further establish the need to incorporate such interaction effect in health care personnel.

## Supplementary material

For supplementary material accompanying this paper visit https://doi.org/10.1017/S1463423620000377. click here to view supplementary material

## Availability of data and materials

Data are available from the corresponding author on reasonable request
