# Supplementary material for: The interaction effect between gender and profession in posttraumatic growth among hospital personnel
Source: Prim Health Care Res Dev. 2020 Sep 24;21:e35. doi: 10.1017/S1463423620000377 (PMC7576536; doi:10.1017/S1463423620000377)

**Electronic Supplementary Material (ESM)**

The following five figures represent the PTG subscales for the interaction of Gender X Profession. All the interactions are significant with p < 0.05 or lower.

**Supplementary Figure 2.** Interaction of Gender X Profession regarding PTG – relating to others subscale.


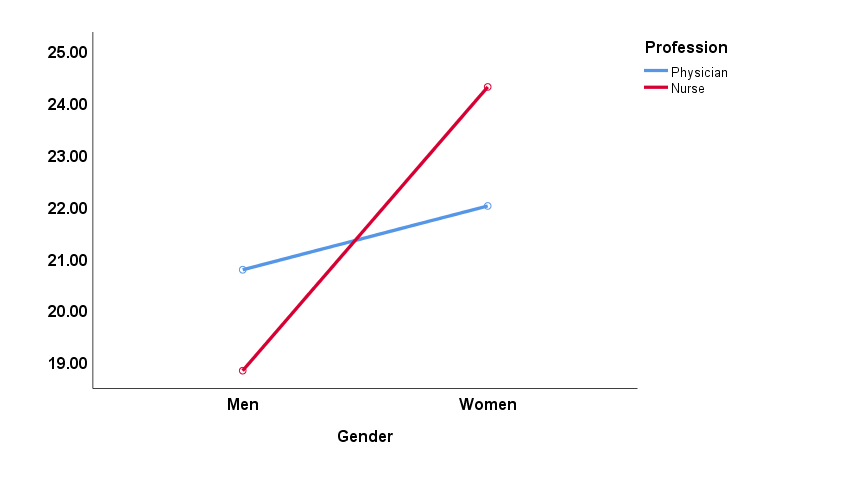


**Supplementary Figure 3.** Interaction of Gender X Profession regarding PTG – new possibilities subscale.


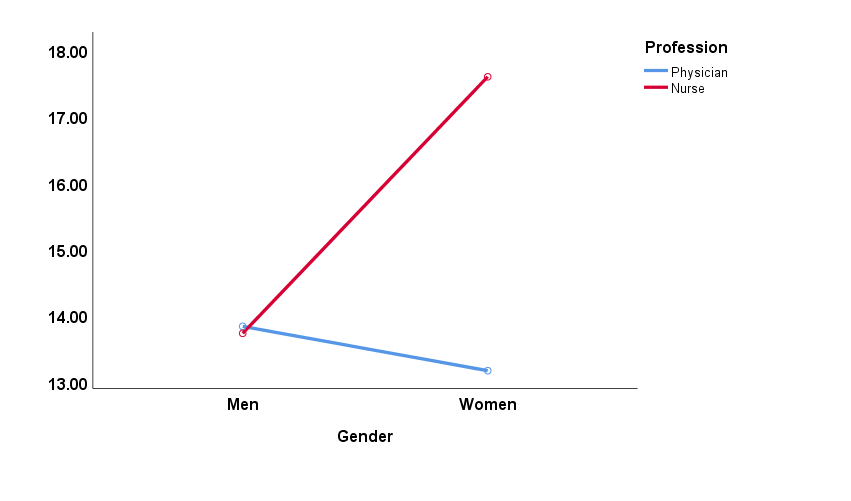


**Supplementary Figure 4.** Interaction of Gender X Profession regarding PTG – personal strength subscale.


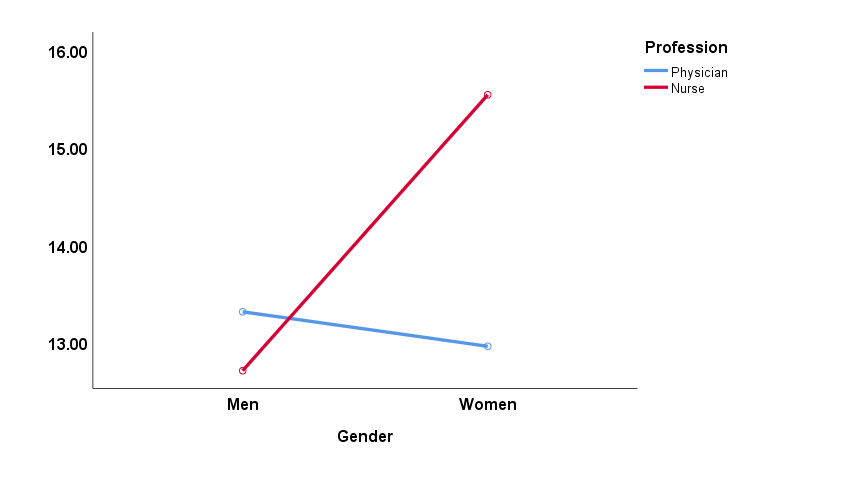


**Supplementary Figure 5.** Interaction of Gender X Profession regarding PTG – spiritual change subscale.


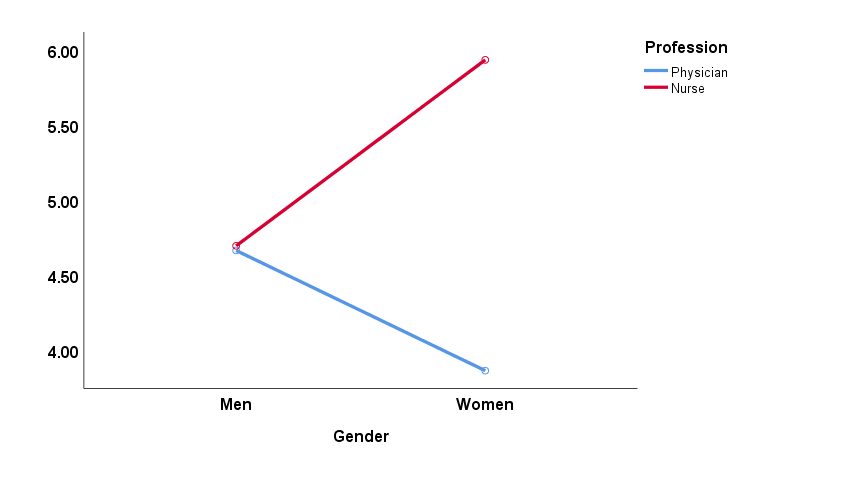


**Supplementary Figure 6**. Interaction of Gender X Profession regarding PTG – appreciation of life subscale.


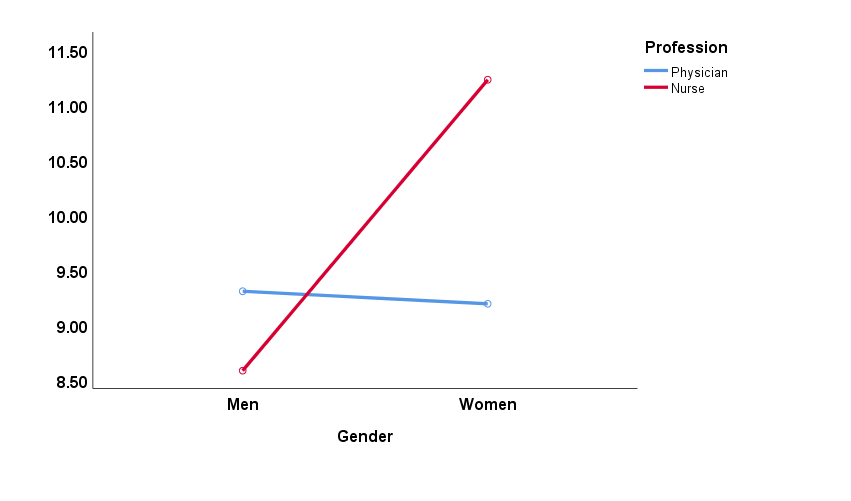

Supplement: Supplementary file 1 [file S1463423620000377sup.zip › S1463423620000377sup002.docx]
